# Pharmacokinetic Interactions Between Antiseizure and Psychiatric Medications

**DOI:** 10.2174/1570159X20666220524121645

**Published:** 2023-06-15

**Authors:** Gaetano Zaccara, Valentina Franco

**Affiliations:** 1 Regional Health Agency of Tuscany, Firenze, Italy;; 2 Department of Internal Medicine and Therapeutics, Clinical and Experimental Pharmacology Unit, University of Pavia, Pavia, Italy;; 3 IRCCS Mondino Foundation, Pavia, Italy

**Keywords:** Antiseizure medications, epilepsy, pharmacokinetic interactions, antidepressants, antipsychotics, anxiolytics

## Abstract

Antiseizure medications and drugs for psychiatric diseases are frequently used in combination. In this context, pharmacokinetic interactions between these drugs may occur. The vast majority of these interactions are primarily observed at a metabolic level and result from changes in the activity of the cytochrome P450 (CYP). Carbamazepine, phenytoin, and barbiturates induce the oxidative biotransformation and can consequently reduce the plasma concentrations of tricyclic antidepressants, many typical and atypical antipsychotics and some benzodiazepines. Newer antiseizure medications show a lower potential for clinically relevant interactions with drugs for psychiatric disease. The pharmacokinetics of many antiseizure medications is not influenced by antipsychotics and anxiolytics, while some newer antidepressants, namely fluoxetine, fluvoxamine and viloxazine, may inhibit CYP enzymes leading to increased serum concentrations of some antiseizure medications, including phenytoin and carbamazepine. Clinically relevant pharmacokinetic interactions may be anticipated by knowledge of CYP enzymes involved in the biotransformation of individual medications and of the influence of the specific comedication on the activity of these CYP enzymes. As a general rule, these interactions can be managed by careful evaluation of clinical response and, when indicated, individualized dosage adjustments guided by measurement of drugs serum concentrations, especially if pharmacokinetic interactions may cause any change in seizure control or signs of toxicity. Further studies are required to improve predictions of pharmacokinetic interactions between antiseizure medications and drugs for psychiatric diseases providing practical helps for clinicians in the clinical setting.

## 1 INTRODUCTION

Psychiatric comorbidities are frequently reported in people with epilepsy (from 25% to 50%) [1, 2]. In subjects with poorly controlled epilepsy, the prevalence of psychosis, mood disorders, and cognitive dysfunction has been reported to be 60% [3]. In such cases, co-prescription of antiseizure medications (ASMs) and drugs for psychiatric diseases are needed. Therefore, there is a potential for pharmacodynamic and pharmacokinetic drug-drug interactions (DDIs) that may alter the effect of a treatment, thus leading to reduced efficacy or increased toxicity.

The characterization of major drug-metabolizing enzymes is performed during preclinical drug development, through *in vitro* and *in vivo* studies. Such studies also allow the identification of inducing or inhibiting properties of the investigational agent on different enzymatic systems involved in the metabolism of drugs, mainly the cytochrome P450 enzyme (CYP) and the uridine diphosphate-glucuronosyltransferase (UGT) [4, 5]. More recently, the effect of many drugs on several transporters that affect the permeability of a wide range of compounds across cell membranes [6, 7] has been assessed in *in vivo* and *in vitro* laboratory preclinical investigations [8]. Such studies have greatly improved the understanding of pharmacokinetic DDIs, thus allowing the prediction of potential DDIs. These findings are stored in large-scale DDI databases, and several drug compendia support DDIs prediction in the clinical setting. Clinical studies in healthy volunteers, studies in patients in whom these DDIs have been studied through a formal protocol and case reports confirm such predictions, although, because of the selection of different doses or different population samples, discrepancies may be found between database predictions and clinical data. In the present review, all potential pharmacokinetic DDIs between ASMs and drugs used for psychiatric diseases have been searched in drug compendia and clinical data for any identified DDI have been searched with a focus on concordances or possible discrepancies.

## 2 SEARCH METHODS AND SELECTION CRITERIA FOR IDENTIFICATION OF DRUG INTERACTIONS

We systematically searched all DDIs between ASMs and drugs that belong to the class of antidepressants (ADs), antipsychotics (APs), and anxiolytics listed according to the Anatomical Therapeutic Chemical (ATC) classification system under N06A, N05A and N05B subgroups codes respectively. Only psychiatric agents marketed in the European Union (EU) or the United States (USA) and for which a summary of product characteristics (SmPC) or Food and Drug Administration (FDA) Prescribing Information (PI) was available were evaluated.

The following ASMs have been included in the search: brivaracetam (BRV), cannabidiol (CBD), carbamazepine (CBZ), cenobamate (CNB), clobazam (CLB), clonazepam (CNP), eslicarbazepine acetate (ESL), ethosuximide (ETS), felbamate (FBM), gabapentin (GBP), lacosamide (LCM), lamotrigine (LTG), levetiracetam (LEV), oxcarbazepine (OXC), perampanel (PER), phenytoin (PHT), phenobarbital (PB), pregabalin (PGB), rufinamide (RFN), stiripentol (STP), topiramate (TPM), valproic acid (VPA), vigabatrin (GVG) and zonisamide (ZNS).

All potential DDIs between ASMs and all drugs of the classes of psychiatric agents mentioned above were searched in publicly accessible drug compendia (Medscape Interaction Checker and RxList) [9, 10] or in the SmPC or FDA PI of each drug. When a potential interaction emerged, the literature was searched for available clinical evidence through MEDLINE (accessed by PubMed: name of the psychiatric agent AND name of each ASM AND drug interaction).

## 3 MECHANISMS OF INTERACTIONS BETWEEN ANTISEIZURE AND PSYCHIATRIC MEDICATIONS

The majority of clinically relevant DDIs between antiseizure and psychiatric drugs occur at the oxidative metabolism level and usually involve the cytochrome CYP system or, to a lesser extent, glucuronidation by UGT or changes in drug distribution across membranes by transmembrane polypeptides, including P-glycoprotein (P-gp) [11, 12].

### 3.1 Drug Interactions Affecting Metabolism

The old-generation ASMs PB, PHT, and CBZ are broad-spectrum strong enzyme inducers as they can induce the activity of many CYP enzymes (particularly CYP3A4, CYP1A2, and CYP2C9) as well as UGT isoenzymes and epoxide hydrolase and by this mechanism can lead to a decrease in blood levels that may result in loss of efficacy of the affected drug. Several second-generation ASMs have weaker enzyme-inducing properties, often limited to some CYP enzymes. This is the case of ESL, OXC, FBM, RFN, TPM at doses higher than 200 mg/day and PER at doses higher than 8 mg/day [13-15]. Other ASMs such as VPA, FBM, STP, CBD and BRV have mainly inhibiting properties and may increase concentrations of the associated drugs [13].

Some ASMs including OXC, STP, FBM and CBD may exert inducing and inhibiting effects on the same or other enzymes [12, 14] and therefore have less predictable effects. In this case, the net result of these DDIs can be either a negligible effect or an increase or a reduction of blood levels of the affected drug. For example, OXC may decrease concentrations of drugs metabolized by CYP3A4, such as PER and increase concentrations of drugs metabolized by CYP2C19, such as VPA. As for ASMs, enzymes that metabolize ADs, APs and anxiolytics also pertain to the CYP system and, to a lesser extent, to the UGT system. Many of these drugs are metabolized by the same CYP or UGT enzymes possibly induced or inhibited by ASMs and sometimes have inhibiting effects on the metabolism of ASMs.

### 3.2 Drug Interactions Affecting Transmembrane Polypeptides

Recently, it has been observed that DDIs may involve several transmembrane polypeptides, including P-gp (permeability glycoprotein also known as multidrug resistance protein MDR1), which transport a wide variety of compounds across cellular membranes, thus influencing their absorption, disposition and elimination [8]. Interestingly, the activity of these proteins may be induced or inhibited [6] leading to changes in the blood and brain concentrations of substrate drugs. Induction of P-gp may affect concentrations and, ultimately, the effect of a substrate drug by reducing its absorption or distribution in the brain or increasing its elimination. The opposite is observed with P-gp inhibition. Several ASMs are inducers (CBZ, PHT, PB), while some of the newer ASMs, such as CBD, STP and BRV, are inhibitors of these transporters [12, 16]. In addition, several ADs and APs [17-19] interact with P-gp as both substrates and inhibitors.

*In vitro* studies and experimental animal studies show that these interactions might have consequences on the efficacy of treatment [17, 20], although no definitive conclusions can yet be drawn. The main mechanisms of elimination and their inducing and/or inhibiting effects on CYP and UGT enzymes and on transporter proteins are reported in Table **1** for ASMs [21], Table **2** for Ads [22], Table **3** for APs, and Table **4** for anxiolytics.

## 4 DRUG-DRUG INTERACTIONS BETWEEN ASMs AND DRUGS FOR PSYCHIATRIC DISEASES

A total of 150 drugs included in the groups N06A, N05A and N05B of the ATC classification system were evaluated. Of those 47 drugs (25 ADs, 16 APs, 6 anxiolytics) with available SmPC and/or FDA PI and information on DDIs with ASMs (from the consultation of drug compendia and/or SmPC/PI of each drug) were selected.

Here, all DDIs between ASMs and ADs (Table **5**), APs (Table **6**), and anxiolytics (Table **7**) will be described. Since CYP2D6, a key isoenzyme contributing to the metabolism of several ADs and APs, does not has a primary role in the metabolism of ASMs and is not induced by these drugs, it will not be discussed.

### 4.1 Drug-Drug Interactions Between ASMs and ADs

For a description of all potential DDIs between ASMs and ADs and a synthesis of clinical findings, see Table **5**.

#### 4.1.1 Older Antidepressants

*Desipramine* is, in part, metabolized and is a weak inhibitor of CYP3A4. Therefore, mild DDIs with ASMs are expected. To date, no clinically relevant DDIs have been described.

*Imipramine* being a CYP1A2, CYP3A4 and CYP2C19 substrate, it is likely that it can be affected by ASMs. In a study conducted in 4 volunteers, co-administration of a barbituric to ongoing treatment with imipramine was associated with a decrease of imipramine plasma levels from 31 to 6 ng/ml [23]. A combination of imipramine and CBZ in 36 children with attention deficit hyperactivity disorder showed significantly lower imipramine levels compared with patients treated with imipramine alone despite receiving larger imipramine doses [24]. Coadministration of CBZ 400 mg/day in 13 patients with major depression treated for three weeks with imipramine resulted in an approximately 50% reduction of imipramine plasma concentration and a slight decrease of its active metabolite, desipramine [25]. Imipramine may inhibit the metabolism of some ASMs [26]. Two case reports described an increase in serum phenytoin levels after imipramine co-administration, possibly caused by CYP2C19 inhibition [27].

*Clomipramine* being a substrate of CYP1A2, CYP3A4, CYP2C19 and of glucuronidation enzymes, may be a victim of DDIs caused by ASMs. Elevation of clomipramine and its active metabolite (desmethyl-clomipramine) concentrations was observed in a 46-year-old female under clomipramine treatment after combination with VPA (1000 mg/day) [28]. Development of status epilepticus in a subject whose seizures were well controlled by VPA after combination with clomipramine (75 mg/day) has been attributed to toxic clomipramine levels and consequent proconvulsant effects caused by the VPA-induced inhibition of CYP2C19 and/or UGT enzymes [29].

*Amitriptyline* metabolism is potentially affected by ASMs because it is partially metabolized by CYP2C19, CYP3A4 and to a lesser extent by CYP1A2 and CYP2C9. The effect of VPA (divalproex sodium) on the pharmacokinetics of amitriptyline and its active metabolite nortriptyline has been investigated in an open-label study conducted in 15 healthy volunteers. The AUC of amitriptyline and its active metabolite nortriptyline levels were significantly increased in subjects treated with VPA [30]. A subsequent retrospective study on a therapeutic drug monitoring (TDM) database confirmed that the combination of amitriptyline with VPA was associated with increased levels of the antidepressant and of nortriptyline compared with matched controls [31]. Other studies have confirmed this DDI [32, 33]. On the opposite, in a similar study on a TDM database, the concentration/daily dose (C/D) ratio of amitriptyline in patients treated with the antidepressant in combination with CBZ was about 50% lower compared with patients receiving amitriptyline alone [34].

*Nortriptyline* is hydroxylated, N-oxidated and conjugated with glucuronic acid. Therefore, even though its metabolism has not been fully investigated, it may interact with ASMs. A case report of a 73-year-old woman, affected by a bipolar manic-depressive disorder, who received nortriptyline (75 mg/day) and CBZ, has been described. Very low levels of nortriptyline, thus requiring a doubling of nortriptyline daily dose (150 mg), were found [35]. Clinical studies and a case report suggest that nortriptyline metabolism may also be inhibited by VPA [32, 36] with a consequent increase in nortriptyline concentrations. In one study, nortriptyline coadministration was found to slightly increase PHT levels [37].

*Doxepin* is hydroxylated, N-oxidated, and conjugated with glucuronic acid and it can be affected by ASMs. In a retrospective study conducted on a TDM database, a combination of VPA with doxepin led to higher doxepin levels compared with doxepin concentrations in subjects not treated with VPA [38].

*Tranylcypromine* metabolism is not known. It is suggested that this drug may increase PB and CBD levels by CYP2C19 inhibition. A case report documented no interaction between tranylcypromine and CBZ [39]^.^

*Mianserin* metabolism includes hydroxylation (mainly by CYP2D), demethylation (primarily by CYP2B6) and oxidation (by CYP1A2 and CYP3A4) [40]. In the compendia, it is reported that CBZ and PB may increase mianserin metabolism. In 4 psychiatric patients, the combination of CBZ and mianserin was associated with a 30% reduction in serum mianserin levels [41]^.^

#### 4.1.2 Newer Antidepressants

*R-citalopram* and *S-citalopram* metabolism can be affected by ASMs because these drugs are CYP2C19 and, to a lesser extent, CYP3A4 substrates. In a pilot clinical study, 6 patients affected by a major depression treated with citalopram (40-60 mg/day) showed a significant decrease in plasma concentrations of R-citalopram and S-citalopram by 31% and 27% respectively after combination with CBZ (200-400 mg/day) for 4 weeks [42].

Citalopram does not affect the metabolism of ASMs. In a study conducted in 12 healthy male subjects, citalopram (40 mg/day) did not change drug levels of CBZ (400 mg/day) or its active metabolite carbamazepine 10,11-epoxide [43].

*Fluoxetine* being a substrate and also a moderate/weak inhibitor of enzymes involved in the metabolism of many ASMs (CYP2C9, CYP2C19 and CYP3A4), may have DDIs with several ASMs. Case reports have described that this agent may significantly increase PHT levels (by CYP2C9 and CYP2C19 inhibition) with possible signs of PHT toxicity [44-46]. Early case reports have also documented inhibition of CBZ metabolism by fluoxetine [47, 48]. In addition, a clinical study has demonstrated that administration of fluoxetine (20 mg/day) in 6 healthy volunteers under treatment with CBZ (400 mg/day) resulted in a significant increase in the AUC of CBZ and of the carbamazepine 10,11-epoxide [47]. No significant changes were demonstrated in a study conducted in eight patients with epilepsy receiving CBZ (800-1600 mg/day) after comedication with fluoxetine (20 mg/day) for 3 weeks [49]. In a series of case reports, a decreased metabolism of VPA with consequently increased levels of the drug has been described in patients co-medicated with fluoxetine, presumably through CYP2C9 inhibition or impaired glucuronide formation [50-52]. In a retrospective study of routine serum concentration measurements of LTG, a 39% lower C/D ratio of LTG was observed when this agent was combined with fluoxetine [53]. This DDI does not have a clear explanation as fluoxetine is not known to have enzyme-inducing properties.

*Fluvoxamine* is a CYP1A2 substrate, and its metabolism may be induced or inhibited by ASMs. On the other hand, fluvoxamine inhibits several CYP enzymes involved in the metabolism of ASMs (mainly CYP2C19, CYP2C9 and CYP3A4). Several case reports have described that this agent increases CBZ levels with consequently increased toxicity through CYP3A4 inhibition [54-56], while CYP2C9 and CYP2C19 inhibition may explain the 3-fold increase in serum concentrations of PHT observed after administration of fluvoxamine in a patient [57].

*Paroxetine*, a CYP3A4 substrate, might be a victim of enzyme-inducing ASMs, although no potential DDIs are described in the compendia. Indeed, in a study of 10 healthy male subjects, it was observed that PB, PHT, and CBZ decrease plasma levels of paroxetine by ∼25% [58].

Paroxetine is a strong CYP2D6 inhibitor, an enzyme not involved in the metabolism of ASMs. Therefore relevant changes in plasma levels of co-administered ASMs are not expected and it has been demonstrated in a placebo-controlled, cross-over study in patients with epilepsy where no significant change in PHT, CBZ or VPA plasma levels after combination with paroxetine was attested [59].

*Sertraline* is predominantly metabolized by CYP2B6 and marginally metabolized by CYP2C19, CYP2C9 and CYP3A4 enzymes. A case-control study described a 3 times lower sertraline C/D ratio with PHT or CBZ [60] while a marked decrease in plasma sertraline levels with consequent sertraline inefficacy was demonstrated in 2 patients receiving CBZ [61]. Since this agent is a weak inhibitor of CYP2C9, CYP2C19 and CYP3A4, it is expected that this drug may potentially inhibit some ASMs. Two double-blind, placebo-controlled studies in healthy volunteers failed to demonstrate a significant effect of sertraline (200 mg/day) on the metabolism of CBZ (400 mg/day) [62] or PHT (300 mg/day) [63]. However, in two elderly patients, sertraline addition to ongoing treatment with PHT resulted in the elevation of PHT concentrations [64]. A report of two clinical cases in which LTG levels were consistently increased with signs of toxicity after combination with sertraline suggests a not predicted DDI between these two drugs that can be consequent to inhibition of LTG glucuronidation by sertraline [65]. However, through a retrospective analysis of a TDM database, it was observed that the combination of sertraline with LTG was associated with a slight and not significant increase in LTG levels [66]. Finally, in a report of a patient with bipolar depression, addition of sertraline to an ongoing treatment with VPA resulted in a 3-fold elevation in VPA levels [67].

*Venlafaxine* is largely metabolized by CYP2D6 and, to a lesser extent, by CYP3A4 and CYP2C9. In 2 retrospective studies conducted on TDM data samples of patients receiving a combination of venlafaxine and VPA were compared with controls without VPA comedication. In both studies, it was observed that while venlafaxine levels were not changed by VPA, there was a significant increase of the dose-corrected serum level of the active venlafaxine metabolite (O-desmethylvenlafaxine) [38] and in the desmethylvenlafaxine/venlafaxine ratio [68]. It has been suggested that the relative elevation in serum concentrations of the pharmacologically active metabolite O-desmethylvenlafaxine may be explained as a consequence of the inhibition of the CYP2C9-mediated N-demethylation of venlafaxine by VPA and acceleration of the O-demethylation.

*Milnacipran* metabolism is partly dependent on CYP3A4 and, therefore, can be slightly affected by several ASMs. In a study in healthy subjects, co-administration with CBZ (400 mg/day) decreased milnacipran levels by approximately 20% [69].

*Duloxetine* is metabolized mainly by CYP1A2 and can be potentially affected by CYP1A2 inducers such as barbiturates, PHT and CBZ. This agent does not affect CYP enzymes involved in the metabolism of ASMs. No clinical data are currently available.

*Trazodone* is a substrate and inhibitor of CYP3A4, and therefore its metabolism can be accelerated by enzyme-inducing ASMs with consequently reduced efficacy, and it may increase levels of all ASMs metabolized by this enzyme. A 53-year-old man with a secondarily generalized partial epilepsy treated with CBZ received trazodone (100 mg/day) and a relevant increase of serum C/D ratio of CBZ with no signs of toxicity was found [70]. In another report of a 77-year-old woman chronically treated with carbamazepine, the addition of trazodone was found to increase CBZ serum concentrations with symptoms of carbamazepine toxicity and to return to baseline with a progressive reduction of toxic symptoms after trazodone discontinuation [71]. These effects may be attributed to CYP3A4 inhibition. In an open-label, randomized, 5-period cross-over trial with single-dose administrations of GBP and trazodone in healthy subjects absence of a pharmacokinetic interaction between the two drugs was demonstrated [72].

*Viloxazine* is a strong CYP1A2 inhibitor and a weak CYP3A4 inhibitor. Although not described in drug compendia, it is expected that this drug may inhibit the metabolism of several ASMs. A 55% increase in CBZ levels with toxic symptoms was found in 6 patients after viloxazine coadministration, possibly due to CYP3A4 inhibition [73, 74]. An increase in PHT levels from a mean value of 18.8 µg/ml to 25.7 µg/ml has also been observed after coadministration with viloxazine to ongoing treatment with PHT in 10 patients with epilepsy [75]. In six patients with epilepsy treated with OXC, administration of viloxazine resulted in an 11% increase in the plasma concentration of the OXC active metabolite 10,11-dihydro-10-hydroxy-carbazepine and a 31% decrease in diol metabolite levels [76], possibly due to inhibition of 10,11-dihydro-10-hydroxy-carbazepine conversion to the inactive diol metabolite.

*Mirtazapine* being partially metabolized by CYP3A4 may have its levels affected by ASMs. Possible effects of this drug on CYP enzymes are not reported in drug compendia. In a study conducted in 24 healthy subjects, co-administration of CBZ (400 mg/day) significantly decreased AUC and C_max_ values of mirtazapine by 61% and 39%, respectively and increased C_max_ values of its active metabolite, demethyl-mirtazapine while mirtazapine did not affect CBZ pharmacokinetic parameters, although it decreased carbamazepine-10,11-epoxide levels [77]. Similarly, a randomized, parallel-group study reported that in 17 healthy subjects, co-administration with PHT (200 mg/day) produced a 47% AUC decrease and a 33% C_max_ decrease of mirtazapine while there was no effect of mirtazapine on PHT metabolism [78].

*Bupropion* is a substrate of CYP2B6, an enzyme slightly induced by several ASMs and is primarily metabolized to hydroxybupropion, an active and potentially toxic metabolite. In a clinical study in patients with mood disorders, pharmacokinetic profiles of bupropion and its metabolites were assessed after single doses (150 mg) of bupropion while receiving placebo or during CBZ or VPA monotherapy. A significant C_max_ and AUC reduction (86% and 90% respectively) of bupropion were observed and the AUC of the active metabolite, hydroxybupropion, was increased by 50% in subjects treated with CBZ while VPA had no effects on bupropion metabolism [79]. This DDI is presumably mediated by the induction properties of CBZ on CYP2B6. An experimental animal study has shown that this agent might have inhibiting properties on PHT metabolism [80], but no clinical studies are available to confirm these findings. Finally, in an open-label, two-way crossover study in healthy volunteers, bupropion (150 mg twice/day) did not cause clinically relevant changes in the pharmacokinetics of a single dose of LTG (100 mg) [81].

*Agomelatine* is primarily metabolized by CYP1A2 (90% of its metabolism) and, to a lesser extent, by CYP2C9 and CYP2C19 (10%) and does not affect CYP enzymes. Therefore, it may be a victim of DDIs from several ASMs. This drug is not reported in drug compendia and no interaction studies or case reports have been described.

*Vilazodone* is predominantly eliminated by CYP3A4. This drug does not affect CYP enzymes involved in the metabolism of ASMs. An open-label study has evaluated the effect of an extended-release formulation of CBZ on the pharmacokinetics of vilazodone (40 mg once daily) in adult healthy subjects and has shown that vilazodone exposure at steady-state decreased by about 45% [82].

*Vortioxetine*, being partially metabolized by CYP3A4, CYP2C19, CYP2C9, and CYP2B6, may be potentially affected by enzyme-inducing ASMs. *In vitro* studies have shown that this agent inhibits CYP2C19, CYP2C9, and CYP2C8 enzymes and, therefore, might affect the clearance of several ASMs [83], but the applicability of these results in the clinical setting remains to be assessed.

### 4.2 Drug-Drug Interactions Between ASMs and APs

For a description of all potential DDIs between ASMs and APs and a synthesis of clinical findings, see Table **6**.

#### 4.2.1 Typical Antipsychotics

*Chlorpromazine* is not metabolized by enzymes that metabolize or are induced or inhibited by ASMs. However, drug compendia indicate a potential induction of its metabolism by CBZ and PB. Indeed, in a study conducted in experimental animals, it has been shown that simultaneous administration of chlorpromazine with CBZ was associated with an increase in the biotransformation of chlorpromazine and reduced biotransformation of CBZ [84]. In the mid-1970, in several case reports, the addition of PB resulted in reduced chlorpromazine levels [85, 86]. Furthermore investigation on VPA pharmacokinetics in schizophrenic patients treated with chlorpromazine suggested that chlorpromazine inhibits the metabolism of VPA [87]. This DDI is not reported in drug compendia.

*Trifluoperazine*, a CYP1A2 substrate, may be induced or inhibited by some enzyme-inducing ASMs, but no clinical data are currently available.

*Haloperidol*, being a CYP3A4 substrate, is potentially induced by all enzyme-inducing ASMs and inhibited by VPA. Several case reports and pharmacokinetic studies have shown that a combination of CBZ with haloperidol decreases plasma haloperidol concentrations by between 20 and 80%, leading to a worsened therapeutic response in patients treated with moderated-dose haloperidol [86, 88-90]. On the contrary, a combination with VPA was not associated with significant changes in haloperidol levels [86, 87, 89]. Levels of haloperidol decanoate, usually administered as an intramuscular injection for long-term treatment of mental disorders, are also reduced by enzyme-inducing ASMs. It has been shown that patients treated with both haloperidol decanoate and enzyme-inducing ASMs, had plasma concentrations measured before the injection significantly lower than those observed in patients not treated with enzyme-inducing ASMs. Consequently, a reduction of the interval between injections has been suggested in order to maintain haloperidol therapeutic plasma concentrations [91]. In a pharmacokinetic study conducted in twelve healthy volunteers, the addition of TPM to haloperidol was associated with slightly increased plasma haloperidol concentrations [92].

#### 4.2.2 Atypical Antipsychotics

*Ziprasidone* is partially metabolized by CYP3A4, it is a weak inhibitor of this enzyme and its metabolism may be induced by enzyme-inducing ASMs. Ziprasidone might also affect the metabolism of some ASMs. A formal parallel-group study in 25 healthy volunteers showed that CBZ was associated with a reduction in ziprasidone exposure (AUC_0-12h_) and C_max_ (36% and 27%, respectively) that was considered not clinically relevant [93].

*Clozapine* being a substrate of CYP1A2 and to a minor extent of CYP3A4, is at high risk of induction by enzyme-inducing ASMs. In a study based on a clozapine TDM database, patients treated with CBZ and clozapine showed a mean C/D ratio of clozapine 50% lower compared with the group of patients on monotherapy [94]. Likewise, Tiihonen *et al.* described a 47% decrease in plasma levels of clozapine in 12 patients co-treated with CBZ compared with those receiving OXC alone because of the lower inducing effect of OXC [95]. In a comparative study in patients with schizophrenia treated with clozapine alone or in combination with PB, patients co-medicated with PB had significantly lower plasma clozapine levels and significantly higher levels of the metabolite clozapine N-oxide because of induction of N-oxidation and demethylation pathways [96]. A more marked DDI has been documented in 2 patients after the addition of PHT to a stable clozapine treatment that led to a decrease of clozapine levels by 65-85% with worsening of psychotic symptoms [97]. The DDI between clozapine and VPA is of special interest and has been investigated in several studies. While in some studies, it has been observed that VPA may inhibit clozapine conversion to norclozapine, in other studies, clozapine levels have been reported to decrease by 41% after VPA addition [22]. The explanation for these controversial results has been given in a study [98] and a case report [99] showing that VPA behaved as a clozapine inhibitor in non-smokers and as an inducer in smokers. More recently, retrospective analyses of patients receiving both clozapine and VPA have shown that the effect of VPA is influenced by smoking and counteracts the inhibitory effects of antidepressants [100] or has inducing effects [101]. It has also been suggested that VPA-induced inhibition of clozapine metabolism increases the risk of myocarditis due to rapid clozapine titration [102, 103] as it happens with the increased risk of serious idiosyncratic reactions due to LTG addition to an ongoing VPA treatment [104]. Finally, although in one study it has been reported that LTG increases clozapine levels and toxicity [105], these data have not been confirmed by subsequent studies [106].

*Olanzapine* is partially metabolized by CYP1A2 and is a substrate of UGT glucuronidation. Several studies showed that CBZ induces olanzapine metabolism. In 11 healthy volunteers, co-administration of CBZ (400 mg/day) with olanzapine resulted in a 34% reduction of olanzapine AUC and a 46% increase in its clearance [107]. A CBZ-induced 36-71% reduction of the median C/D ratio of olanzapine has been subsequently documented in several studies [22]. A predicted DDI between VPA and olanzapine (both compounds are at least partially glucuronized by UGT1A4) has been investigated in several studies with controversial results. Case reports and clinical studies in patients with bipolar or schizoaffective disorders have described that combination with VPA produces no significant effect or decreased levels of olanzapine [22]. It has been suggested that VPA may act both as an inducer and a competitive inhibitor of olanzapine metabolism depending on VPA concentration and the smoking status of evaluated patients [22, 108]. More recently, in a study on a large database, it has been clarified that concurrent use of VPA significantly decreases serum concentrations of olanzapine to an extent that is related to smoking status [109]. In addition, it has been shown that VPA has no effect on olanzapine concentrations when given in patients treated with a long-acting injectable formulation of olanzapine. It was concluded that the mechanism involved in this interaction was restricted to oral olanzapine treatment [110]. This effect of VPA on olanzapine clearance has been recently confirmed in a large retrospective study [111]. Being olanzapine glucuronized by UGT1A4, which is also involved in LTG metabolism, a DDI between these two drugs has been hypothesized. Indeed, two studies in healthy volunteers found no effects of LTG on olanzapine levels [112, 113], while in one study in patients, it has been found that LTG has a mild effect of increasing olanzapine concentrations at doses higher than 200 mg/day [114].

*Quetiapine* is mainly metabolized by CYP3A4 and although this drug is not included in drug compendia, it may be anticipated that quetiapine can be a victim of several DDIs with ASMs.

In a study conducted in 18 patients with psychiatric disorders and treated with quetiapine (300 mg/day), the addition of CBZ (600 mg/day) decreased quetiapine C_max_ by 80% with a relative increase of its oral clearance of about 7.5 folds [115]. Such findings have been confirmed by several retrospective studies conducted on TDM databases [116-118]. In the study conducted by Castberg *et al.*, co-administration of quetiapine with CBZ was associated with an 86% decrease in C/D ratio of quetiapine compared to patients on quetiapine monotherapy [117]. There is also evidence that PHT 300 mg/day decreased plasma levels of quetiapine 750 mg/day by approximately 80% in patients with schizophrenia, schizoaffective disorder or bipolar disorder as a result of a strong inducing effect of phenytoin on quetiapine biotransformation mediated by CYP3A4 [119]. These changes are explained by the inducing properties of CBZ and PHT on CYP3A4-mediated quetiapine biotransformation. Less consistent findings have been observed in studies assessing the effect of VPA on quetiapine. Although in a study on a TDM database, the quetiapine C/D ratio was significantly higher (77%) in patients in whom quetiapine was combined with VPA compared with patients taking quetiapine alone [120], other two TDM studies [116, 117] and a formal kinetic study [121] did not find significant differences among patients treated with quetiapine alone or in combination with VPA. The interaction between quetiapine and LTG was investigated in two studies on large TDM databases and showed a small but significant decrease in quetiapine C/D ratio when this drug was co-administered with LTG [117, 122]. It has been suggested that these pharmacokinetic changes may be explained by a weak inducing effect of LTG on UGT1A4 glucuronidation that might be involved in quetiapine metabolism. No significant pharmacokinetic interactions have been documented between TPM (200 mg/day) and quetiapine [123]. Interestingly, in two patients in whom this drug had been added to CBZ, it was observed a marked increase of CBZ-10,11-epoxide, the CBZ active metabolite, and associated toxic signs, which returned to baseline levels after quetiapine discontinuation. It has been suggested that, in this case, quetiapine may have inhibited the epoxide hydrolase and glucuronidation of carbamazepine-10,11-trans-diol [124].

*Asenapine* metabolism may be a victim of ASMs because this agent is a substrate of CYP1A2 and, to a minor extent, of CYP3A4 and undergoes glucuronidation by UGT1A4. In a randomized, crossover study in 24 healthy volunteers, VPA 1000 mg/day reduced the formation of the inactive metabolite N-glucuronide without affecting asenapine AUC. It was concluded that VPA inhibits asenapine glucuronidation without significantly affecting asenapine pharmacokinetics [125].

*Risperidone*, being partly metabolized by CYP3A4, can be induced or inhibited by several ASMs. Case reports confirm the predicted induction of metabolism of risperidone by CBZ [126], and in a clinical study, the sum of the concentrations of risperidone and its active metabolite 9-OH-risperidone in patients co-medicated with CBZ was significantly lower compared with patients treated with risperidone alone or in patients receiving combination therapy with VPA [127]. No changes in risperidone and its active metabolite levels have been found in patients evaluated with or without VPA [127, 128]. Although in a case report, it was observed a marked elevation of risperidone levels associated with adverse effects after LTG addition [129], in a prospective study in 10 psychotic patients treated with risperidone (3-6 mg/day), LTG (200 mg/day) did not affect risperidone levels [114]. Instead, a DDI has been observed between risperidone and TPM that, at high doses, is a weak CYP3A4 inducer. In a study in healthy volunteers, TPM (200 mg/day) decreased the AUC of risperidone, given as a single dose of 2 mg, by 23% and increased risperidone clearance by 51% [130]. Some clinical findings suggest that risperidone may have a mild inhibitory effect on CYP3A4. In a study of 8 patients with epilepsy and behavioral disturbances, the combination of risperidone (1 mg/day) with a previous CBZ treatment resulted in a slight increase in CBZ levels [131].

*Aripiprazole* is partly metabolized by CYP3A4 and may be subjected to DDIs by ASMs. In two studies conducted in patients with schizophrenia or schizoaffective disorders, co-administration of CBZ resulted in a significant decrease in the plasma concentration of aripiprazole and dehydroaripiprazole (64% and 68%, respectively) [132] and a significant reduction in aripiprazole mean peak plasma concentration and AUC (66% and 71% respectively) [133]. Studies showing that VPA may have mild inducing effects on aripiprazole metabolism are available. In 10 patients with schizophrenia, VPA co-administration decreased both C_max_ and AUC of aripiprazole by 26% and 24%, respectively [134]. Furthermore, in a TDM study, the aripiprazole C/D ratio was 24% lower in patients co-medicated with VPA compared with patients on aripiprazole monotherapy [135]. It was suggested that these changes might be attributed to VPA mild inducing effects on CYP3A4 and P-gp. Finally, no significant effects of LTG on aripiprazole levels have been observed in a TDM database [136]. Aripiprazole does not affect ASM plasma levels and in an open-label, single-sequence study in healthy volunteers, aripiprazole had no effect on VPA metabolism [137].

*Paliperidone* is predominantly eliminated by renal excretion and only to a minor extent by the CY3A4 enzyme with no relevant effects on CYP enzymes. Consequently, ASMs should not influence in appreciable amount its metabolism. However, in six schizophrenic patients undergoing treatment with paliperidone (6-12 mg/day), concomitant treatment with CBZ 600 mg/day induced a 66% mean reduction of paliperidone levels [138]. In a larger study in 64 patients with schizophrenia, co-administration with CBZ (400 mg/day) was associated with a 37% decrease in paliperidone total exposure (AUC_24 h_) [139]. It has been suggested that this DDI is probably the result of renal P-gp induction by CBZ and a consequence of CYP3A4 induction [22]. The effect of VPA on paliperidone levels has been studied in healthy volunteers treated with repeated doses of VPA (divalproex sodium extended-release) for 18 days (1000 mg/day). The oral bioavailability of a single dose of an extended-release formulation of paliperidone was increased by 51%. No effects on VPA plasma levels were detected in patients with psychiatric disorders treated with multiple doses of paliperidone extended-release [140].

Newer APs including *cariprazine* and *lurasidone* (metabolized by CYP3A4), *pimozide* (partly metabolized by CYP3A4 and, to a lesser extent, by CYP1A2), *iloperidone* and *brexpiprazole* (partly metabolized by CYP3A4) are expected to be potentially affected by several ASMs, but no clinical data are currently available.

### 4.3 Drug-Drug Interactions Between ASMs and Anxiolytics

There is evidence that many benzodiazepines, anxiolytic agents primarily metabolized by CYP3A4, may be affected by enzyme-inducing ASMs. However, given the wide therapeutic index of these drugs, the clinical value of these interactions is limited [86]. CLB and CNP are not included in this list because they are used mainly as ASMs. For a description of all potential DDIs between ASMs and anxiolytics, see Table **7**. No effects of anxiolytic drugs on ASMs have been described.

*Diazepam* is a CYP3A4 and CYP2C19 substrate, and therefore, its metabolism is potentially affected by several ASMs. In some studies conducted more than 20 years ago, it was observed that diazepam and other benzodiazepines induce the metabolism of PB [141]. CBZ has been reported to induce the conversion of diazepam to desmethyldiazepam. This interaction may not necessarily lead to a decreased clinical response as desmethyldiazepam is a pharmacologically active compound [142, 143]. In a study conducted in healthy volunteers, intravenous diazepam (10 mg) was given before and 5 days after a VPA treatment (1500 mg/day). The concentration of unbound diazepam in serum was significantly higher during VPA administration, and mean serum levels of the active metabolite N-desmethyldiazepam were significantly lower, thus suggesting that VPA displaces diazepam from plasma protein binding sites and inhibits its metabolism [144].

*Alprazolam* is a CYP3A4 substrate, and its metabolism may be affected by ASMs. The effect of CBZ (300 mg/day for 10 days) on a single oral dose of alprazolam (0.8 mg) has been investigated in a double-blind, crossover study involving 7 healthy volunteers. Alprazolam oral clearance was increased and the elimination half-life was significantly shortened [145]. Furthermore, a case report showed that the CBZ-induced decrease in alprazolam plasma levels resulted in a clinical deterioration [146].

*Oxazepam* is mainly eliminated by conjugation with glucuronic acid and also these enzymes can be affected by ASMs. The pharmacokinetics of oxazepam has been studied in 9 patients with epilepsy treated with PB and PHT or PHT alone and in 9 healthy subjects, and it has been found that oxazepam elimination half-life was reduced and oral clearance increased in patients compared with matched healthy controls. Oxazepam binding to serum proteins (about 93%) was not affected [147].

*Midazolam* is a CYP3A4 substrate and its plasma concentrations have been found to be markedly reduced after a single oral dose of midazolam (15 mg) in 6 patients receiving CBZ and PHT compared with 7 control subjects, as a consequence of CYP3A4-mediated induction of first-pass metabolism in the liver [148].

*Chlordiazepoxide, clorazepate* and *buspirone* are CYP3A4 substrates while *lorazepam* is metabolized by conjugation with glucuronic acid. Metabolism of all these compounds can be altered by several ASMs. To date no clinical studies or case reports have investigated DDIs between these drugs and ASMs.

## CONCLUSION

Whether drugs used for the treatment of epilepsies and psychiatric disorders exert effects that cannot be fully anticipated, even more difficult is the prediction of the effect of a drug combination. Although not discussed here, other mechanisms, including pharmacodynamic interactions, might play an important role as a source of clinical variability of the effect of drug combinations.

There are several factors that limit the validity of a prediction. Despite the fact that the propensity of a drug to cause a DDI can be predicted by the knowledge of its effects on all CYP and UGT isoenzymes that metabolize the affected drug, the degree of the interaction is subjected to high variability. Several factors, such as drug dose, genetic background and other pharmacokinetic mechanisms may influence the degree of interaction. Treatment strategy selection can be facilitated by knowledge of potential DDIs and underlying mechanisms. However, there are several limits and discrepancies as not all drugs are present in drug compendia and there are cases in which information in the SmPC or PI of a drug is not identical to information from drug compendia. There are also discrepancies between *in vitro* data and results derived from pharmacokinetic studies and even between clinical findings. Adverse clinical consequences may be minimized by individualized dosage adjustments guided by careful evaluation of clinical response and, when indicated, by measurement of serum drug concentrations. In fact, the combination of agents with the potential for pharmacokinetic interactions represents one of the main indications for TDM [149]. Some examples of possible clinical consequences with emphasis on the appearance of adverse effects are summarized in Table **8**.

Further well-designed studies are needed to improve predictions of DDIs. Clinicians should be aware of the importance of DDIs and should pay attention to all factors that influence the degree of interaction when two or more drugs are combined.

## Figures and Tables

**Table 1:** Mechanisms of elimination of antiseizure medications and their inducing and/or inhibiting effects on metabolism enzymes and P-gp.

**Antiseizure Medication**	**The Main Route(s) of Elimination and ** **Transporter Proteins Involved in the ** **Distribution**	**Possible Mechanisms of Interactions. ** **Effects on CYP, UGT and P-gp**	
		**Induction**	**Inhibition**
**Old-Generation Antiseizure Medications**			
Carbamazepine	CYP3A4	Inducer of CYP3A4, CYP2C9, CYP1A2, CYP2B6 and UGT Inducer of P-gp	-
Clobazam	CYP3A4 and CYP2C19	-	-
Clonazepam	CYP3A4	-	-
Ethosuximide	CYP3A4	-	-
Phenytoin	CYP2C9, CYP2C19, CYP2C18 and CYP3A4 P-gp	Inducer of CYP3A4, CYP2C9, CYP1A2 and UGT Inducer of P-gp	Inhibitor of CYP2C19
Phenobarbital	CYP2C9 and CYP2C19 P-gp	Inducer of CYP3A4, CYP2C9 and CYP1A2 Inducer of P-gp	-
Valproic acid	CYP2A6, CYP2C9, CYP2C19, CYP2B6 and mitochondrial oxidases UGT1A3 and UGT2B7	-	Inhibitor of CYP2C9, epoxide hydrolase and UGT enzymes Mild inhibitor of CYP2C19 and CYP3A4
**New Generation Antiseizure Medications**			
Brivaracetam	CYP2C19	Mild inducer of CYP2B6 and CYP3A4	Inhibitor of epoxide hydrolase Mild inhibitor of CYP2C19
Cannabidiol	CYP2C19 and CYP3A4 UGT1A7, UGT1A9 and UGT2B7	Inducer of CYP1A2 and CYP2B6	Inhibitor of CYP1A2, CYP2B6, CYP2C8, CYP2C9 and CYP2C19 Inhibitor of UGT1A9 and UGT2B7 Inhibitor of P-gp and BCRP
Eslicarbazepine acetate^a^	UGT1A4, UGT1A9, UGT2B4, UGT2B7 and UGT2B17 P-gp	Inducer of CYP3A4 Inducer of UGT1A4 Inducer of P-gp	Mild inhibitor of CYP2C19
Felbamate	CYP3A4 and CYP2E1 P-gp	Mild inducer of CYP3A4	Inhibitor of CYP2C19
Gabapentin	Renal excretion	-	-
Lacosamide	CYP3A4, CYP2C9 and CYP2C19	-	-
Lamotrigine	UGT1A4 P-gp	-	-
Levetiracetam	Renal excretion Enzymatic hydrolysis (type-B esterase)	Mild inducer of CYP2B6 and CYP3A4	-
Midazolam	CYP3A4	-	-
Oxcarbazepine^b^	UGT1A4, UGT1A9, UGT2B4, UGT2B7 and UGT2B17	Inducer of CYP3A4 Inducer of UGT1A4 Inducer of P-gp	Mild inhibitor of CYP2C19
Perampanel	CYP3A4	Mild inducer of CYP2B6 and CYP3A4/5	Mild inhibitor of CYP2C8 Mild inhibitor of UGT1A9
Pregabalin	Renal excretion	-	-
Rufinamide	Carboxylesterases	Inducer of CYP3A4	-
Stiripentol	CYP1A2, CYP2C19, CYP3A4 and carboxylesterases	Inducer of CYP3A4	Inhibitor of CYP1A2, CYP3A4, CYP2C19 and CYP2D6 Inhibitor of P-gp and BCRP
Topiramate	Renal excretion Oxidation P-gp	Mild inducer of CYP3A4 (>200 mg/day)	Mild inhibitor of CYP2C19
Vigabatrin	Renal excretion	-	-
Zonisamide	CYP3A4 and N-acetyl transferase	-	-

**Abbreviations**: CYP=cytochrome P450, UGT=Uridine diphosphate-glucuronosyltransferase, P-gp=P-glycoprotein efflux transporter, BCRP=Breast Cancer Resistance Protein.

**Note**: ^a^Eslicarbazepine acetate is a prodrug and is primarily converted to eslicarbazepine. The reported enzymes involved in the elimination process refer to eslicarbazepine.

^b^Oxcarbazepine is a prodrug converted to the active metabolite licarbazepine (racemic mixture of (R)-licarbazepine and eslicarbazepine). The reported enzymes involved in the elimination process refer to licarbazepine.

For a source of references, see Patsalos *et al.* [4]; Patsalos and Perucca [5]; Patsalos, [13, 14]; Zaccara and Perucca [15]; Italiano and Perucca [21] and SmPC or PI of each ASM.

**Table 2:** Mechanisms of elimination of antidepressant drugs, their active metabolites and their effects on metabolism enzymes and P-gp.

**Antidepressant**	**The Main Route(s) of Elimination and Transporter Proteins Involved in the Distribution**	**Possible Mechanisms of Interactions**	
		**Inducing or Inhibiting Effects on CYP, UGT and P-gp**	**Active Metabolite**
**Older Antidepressants**			
Desipramine	Renal excretion (70%) CYP2D6, CYP3A4	Weak CYP3A4 inhibitor	-
Imipramine	CYP1A2, CYP3A4 and CYP2C19 P-gp	P-gp inhibitor	Desmethylimipramine
Clomipramine	CYP1A2, CYP3A4, CYP2C19 and glucuronidation P-gp	-	Desmethylclomipramine
Amitriptyline	CYP2C19, CYP3A4, CYP2D6 and, to a lesser extent, CYP1A2 and CYP2C9 P-gp	P-gp inhibitor	Nortriptyline
Nortriptyline	Hydroxylation (possibly to active metabolites), N-oxidation and glucuronidation P-gp	P-gp inhibitor	Possible active metabolites
Doxepin	Demethylation, N-oxidation, hydroxylation and glucuronidation	-	Desmethyldoxepin
Tranylcypromine	Breakdown of the side chain and probably conjugation	-	-
Mianserin	Aromatic hydroxylation, N-oxidation and N-demethylation and glucuronidation	-	-
**Newer Antidepressants**			
Citalopram	CYP2C19 and, to a lesser extent, CYP3A4 and CYP2D6 P-gp	CYP2D6 (weak)	-
Escitalopram (S-citalopram)	CYP2C19 and, to a lesser extent, CYP3A4 and CYP2D6	CYP2D6 (weak)	-
Fluoxetine	CYP2D6 and, to a lesser extent, CYP2C9, CYP2C19, CYP3A4 P-gp	CYP2D6 strong inhibitor CYP2C9 moderate inhibitor CYP2C19 and CYP3A4 weak to moderate inhibitor	Norfluoxetine
Fluvoxamine	CYP1A2 and CYP2D6 P-gp	CYP1A2 and CYP2C19 strong inhibitor CYP2C9 and CYP3A4 moderate inhibitor CYP2D6 weak inhibitor	-
Paroxetine	CYP2D6 and CYP3A4 P-gp	CYP2D6 strong inhibitor CYP1A2, CYP2C9, CYP2C19 and CYP3A4 weak inhibitor P-gp inhibitor	-
Sertraline	CYP2B6 and, to a lesser extent, CYP2C19, CYP2C9, CYP2D6 and CYP3A4 P-gp	CYP2D6 weak to moderate inhibitor CYP1A2, CYP2C9, CYP2C19 and CYP3A4 weak inhibitor P-gp inhibitor	-
Trazodone	CYP3A4	-	m-chlorophenylpiperazine
Viloxazine	Renal excretion CYP2D6, UGT1A9, and UGT2B15	CYP1A2 strong inhibitor CYP2D6 and CYP3A4 weak inhibitor	-
Mirtazapine	CYP2D6, CYP3A4 and, to a lesser extent, CYP1A2 and UGTs	-	Demethylmirtazapine
Bupropion	CYP2B6	CYP2D6 strong inhibitor	Hydroxybupropion Threohydrobupropion Erythrohydrobupropion
Venlafaxine	CYP2D6 and CYP3A4 P-gp	-	O-desmethylvenlafaxine
Milnacipran	Renal excretion and, to a lesser extent, UGT and CYP3A4	CYP3A4 weak inhibitor	-
Reboxetine	CYP3A4	CYP2D6 and CYP3A4 mild inhibitor	-
Duloxetine	CYP1A2, and, to a lesser extent CYP2D6, and glucuronidation	Moderate CYP2D6 moderate inhibitor P-gp inhibitor	-
Agomelatine	CYP1A2 and, to a lesser extent, CYP2C9 and CYP2C19	-	-
Vilazodone	CYP3A4, and to a lesser extent, CYP2C19, CYP2D6 and carboxylesterase	CYP2C19, CYP2D6 and CYP2C8 moderate inhibitor	-
Vortioxetine	CYP2D6, and, to a lesser extent, CYP3A4, CYP2C19, CYP2C9, CYP2A6, CYP2C8, and CYP2B6	-	-

**Note**: Antidepressants are listed in the same order they appear in the ATC system. For a source of references, see Akamine *et al.* [17], O'Brien *et al.* [20], Spina *et al.* [22] and SmPC or PI of each AD.

**Abbreviations**: CYP=cytochrome P450, UGT= Uridine diphosphate-glucuronosyltransferase, P-gp=P-glycoprotein efflux transporter.

**Table 3:** Mechanisms of elimination of antipsychotic drugs, their active metabolites and their effects on metabolism enzymes and P-gp.

**Antipsychotic**	**The Main Route(s) of Elimination and ** **Transporter Proteins Involved in the ** **Distribution**	**Possible Mechanisms of Interactions**	
		**Inducing or Inhibiting Effects on CYP, UGT and P-gp**	**Main Active Metabolite**
Chlorpromazine	CYP2D6 P-gp	CYP2D6 inhibitor	7-Hydroxychlorpromazine
Trifluoperazine	CYP1A2	-	N-oxide trifluoperazine possibly active
Haloperidiol	CYP3A4 and CYP2D6 P-gp	CYP2D6 weak inhibitor P-gp substrate	Active metabolites not clinically relevant
Ziprasidone	CYP3A4 and aldehyde oxidase	CYP2D6 and CYP3A4 weak inhibitor	**-**
Lurasidone	CYP3A4	-	**-**
Paliperidone	Renal excretion CYP2D6 and CYP3A4 minimally involved	-	**-**
Pimozide	CYP3A4 and, to a minor extent, CYP1A2 and CYP2D6	P-gp substrate	**-**
Clozapine	CYP1A2 and, to a minor extent, CYP3A4 P-gp	-	**-**
Olanzapine	CYP1A2 and CYP2D6 and UGT P-gp	P-gp inhibitor	N-desmethyl and 2-hydroxymethyl metabolites (low clinical relevance)
Quetiapine	CYP3A4 P-gp	CYP1A2, CYP2C9, CYP2C19, CYP2D6 and CYP3A4 weak inhibitor	Norquetiapine
Asenapine	CYP1A2 and, to a minor extent, CYP2D6 and CYP3A4, and UGT1A4	Weak CYP2D6 inhibitor	-
Sulpiride	Mainly cleared unchanged in urine	-	-
Risperidone	CYP2D6 and, to a lesser extent, CYP3A4 P-gp	P-gp inhibitor	9-Hydroxy-risperidone (paliperidone)
Aripiprazole	CYP3A4 and CYP2D6 P-gp	-	Dehydroaripiprazole
Paliperidone	Renal excretion and, to a minor extent, CYP2D6 and CYP3A4 P-gp	-	-
Iloperidone	CYP3A4 and CYP2D6	Weak CYP3A4 inhibitor	P88 and P95
Cariprazine	CYP3A4 and, to a minor extent, CYP2D6	P-gp inhibitor	Desmethyl cariprazine and didesmethyl cariprazine
Brexipiprazole	CYP3A4 and CYP2D6	-	-

**Note**: Antipsychotics are listed in the same order they appear in the ATC system. For a source of references, see Akamine *et al.* [17], O'Brien *et al.* [20], Spina *et al.*, [22] and SmPC of each AP.

For abbreviations, see Table **2**.

**Table 4:** Mechanisms of elimination of anxiolytic drugs, their active metabolites and their effects on metabolism enzymes and P-gp.

**Anxiolytics**	**Maine Route(s) of Elimination**	**Possible Mechanisms of Interactions**	
		**Inducing or Inhibiting Effects on CYP, UGT and P-gp**	**Main Active Metabolite**
Diazepam	CYP3A4 and CYP2C19 Conjugation of its active metabolites with glucuronic acid	-	Desmethyldiazepam, oxazepam and temazepam
Chlordiazepoxide	CYP3A4	-	Desmethylchlordiazepoxide, demoxepam, desmethyldiazepam and oxazepam
Oxazepam	Conjugation with glucuronic acid	-	-
Clorazepate	CYP3A4	-	Nordiazepam (further metabolized by hydroxylation to oxazepam)
Lorazepam	Conjugation with glucuronic acid	-	-
Alprazolam	CYP3A4	-	-
Buspirone	CYP3A4	-	1-(2-pyrimidinyl)-piperazine

**Note**: Anxiolytics are listed in the same order they appear in the ATC system. For a source of references, see the SmPC or PI of each anxiolytic.

For abbreviations, see Table **2**.

**Table 5:** Potential drug interactions between antiseizure medications and antidepressants and synthesis of results of available clinical studies and case reports.

**Antidepressants**	**Effects of Antiseizure Medications on the Antidepressant Drug ** **(Affected Drug)**			**Effects of the Antidepressant Drug ** **(Perpetrator) on Antiseizure Medications**	
**Older Antidepressants**				
**Desipramine**	**Information from Drug Compendia**			
	CYP3A4 induction		CYP3A4 inhibition	
	Carbamazepine Phenytoin Phenobarbital Eslicarbazepine acetate Oxcarbazepine Rufinamide Topiramate	↓↓ ↓↓ ↓↓ ↓ ↓ ↓ ↓	Cannabidiol	↑↑
	No DDIs studies with ASMs in humans or case reports have been described			
**Imipramine**	**Information from Drug Compendia**			
	CYP1A2 and CYP3A4 induction or by CYP3A4 and CYP2C19 inhibition		No predicted effects	
	Carbamazepine Phenytoin Phenobarbital Rufinamide Topiramate Cannabidiol Felbamate Stiripentol Oxcarbazepine	↓↓ ↓↓ ↓↓ ↓ ↓ ↑↑ ↑↑ ↑↑ ↓↑	-	
	Clinical data confirm the induction of imipramine by barbiturates and carbamazepine. Case reports of inhibition of phenytoin metabolism by imipramine have been described			
**Clomipramine**	**Information from Drug Compendia**			
	CYP3A4 induction and CYP2C19 or CYP1A2 induction/inhibition		No predicted effects	
	Carbamazepine Phenytoin Phenobarbital Rufinamide Topiramate Eslicarbazepine acetate Oxcarbazepine Stiripentol Cannabidiol	↓↓ ↓↓ ↓↓ ↓ ↓ ↓↑ ↓↑ ↑ ↑	-	
	Case reports indicate a strong inhibition of clomipramine metabolism by valproic acid (not reported)			
**Amitriptyline**	**Information from Drug Compendia**			
	CYP3A4 induction, CYP2C19 induction/inhibition or P-gp induction (distribution in the SNC)		No predicted effects	
	Carbamazepine Phenytoin Phenobarbital Rufinamide Oxcarbazepine Felbamate	↓↓ ↓↓ ↓↓ ↓ ↓↑ ↑↑	-	
	Pharmacokinetic and retrospective studies have shown that amitriptyline metabolism is induced by carbamazepine and inhibited by valproic acid			
**Nortriptyline**	**Information from Drug Compendia**			
	CYP1A2 and CYP3A4 induction or inhibition		No predicted effects	
	Carbamazepine Valproic acid (from SmPC)	↓ ↑	-	
	Clinical studies and a case report confirm that nortriptyline metabolism is induced by carbamazepine and inhibited by valproic acid			
**Doxepin**	**Information from Drug Compendia**			
	CYP3A4 inhibition		No predicted effects	
	Stiripentol	↑↑	-	
	Findings from a retrospective study have indicated that doxepin metabolism may be inhibited by valproic acid			
	No DDIs studies with ASMs in humans or case reports have been described			
**Tranylcypromine**	**Information from Drug Compendia**			
	No predicted effects		CYP2C19 inhibition	
	-		Phenobarbital Cannabidiol	↑↑ ↑↑
	A clinical case report has shown a lack of DDI with carbamazepine			
**Mianserin**	**Information from Drug Compendia**			
	Unknown mechanism		No predicted effects	
	Carbamazepine Phenobarbital	↓↓ ↓↓	-	
	No DDIs studies in humans or case reports have been described			
**Newer Antidepressants**				
**Citalopram**	**Information from Drug Compendia**			
	CYP3A4 and CYP2C19 induction and/or inhibition		No predicted effects	
	Carbamazepine Phenytoin Phenobarbital Cenobamate Stiripentol Cannabidiol	↓↓ ↓↓ ↓↓ ↓↓ ↓↑ ↑	-	
	A clinical study has confirmed a moderate inducing effect of carbamazepine on citalopram metabolism			
**Escitalopram ** **(S-citalopram)**	**Information from Drug Compendia**			
	CYP3A4 and CYP2C19 induction and/or CYP2C19 inhibition		No predicted effects	
	Carbamazepine Phenytoin Phenobarbital Cenobamate Stiripentol Cannabidiol Felbamate	↓↓ ↓↓ ↓↓ ↓↓ ↓↓↑↑ ↑↑ ↑↑	-	
	No clinical data available (see citalopram)			
**Fluoxetine**	**Information from Drug Compendia**			
	CYP3A4 induction or CYP2C9 inhibition		CYP2C9 or CYP2C19 inhibition	
	Carbamazepine Cannabidiol	↓↓ ↑↑	Phenytoin Cannabidiol	↑↑ ↑↑
	Several case reports and studies on humans have confirmed that phenytoin, carbamazepine and valproic acid metabolism is inhibited by fluoxetine (not predicted by compendia)			
**Fluvoxamine**	**Information from Drug Compendia**			
	CYP1A2 inhibition		CYP3A4 or CYP2C9 inhibition	
	Stiripentol	**↑↑**	Carbamazepine Phenytoin Cannabidiol Zonisamide	↑↑ ↑↑ ↑↑ ↑↑
	Inhibition of carbamazepine and phenytoin levels by fluvoxamine has been observed in case reports (not predicted by compendia)			
**Paroxetine**	**Information from Drug Compendia**			
	No predicted effects		No predicted effects	
	In a study on healthy volunteers, it has been observed that phenobarbital, phenytoin and carbamazepine decreased by 25% paroxetine levels while in a double-blind, cross-over study in patients with epilepsy, phenytoin, carbamazepine or valproate plasma levels were not affected by paroxetine			
**Sertraline**	**Information from Drug Compendia**			
	CYP2C19 inhibition		CYP3A4 inhibition	
	Cannabidiol Cenobamate Stiripentol	**↑↑** **↑↑** **↑↑**	Carbamazepine Phenytoin Cannabidiol	↑↑ ↑↑ ↑↑
	A case report showed that sertraline could be induced by carbamazepine. Two controlled studies did not confirm that sertraline affects the metabolism of carbamazepine or phenytoin, while in case reports, inhibition of phenytoin or valproic acid metabolism by sertraline has been observed			
**Trazodone**	**Information from Drug Compendia**			
	CYP3A4 induction or induction/inhibition		CYP3A4 inhibition and unspecified mechanisms	
	Carbamazepine Oxcarbazepine Eslicarbazepine Phenobarbital Rufinamide Topiramate Stiripentol	↓↓ ↓↓ ↓↓ ↓↓ ↓↓ ↓↓ ↓↑	Carbamazepine Phenytoin	↑↑ ↑
	Trazodone-induced increase in carbamazepine levels has been described in a case report. Lack of DDI between trazodone and gabapentin has been demonstrated in a formal DDI study			
**Viloxazine**	No potential DDIs are reported in drug compendia			
	Studies in patients with epilepsy have documented that viloxazine significantly inhibits the metabolism of carbamazepine and phenytoin and slightly increases concentrations of the oxcarbazepine active metabolite			
**Mirtazapine**	**Information from Drug Compendia**			
	CYP3A4 induction or CYP3A4 and CYP1A2 induction/inhibition		No predicted effects	
	Carbamazepine Phenytoin Phenobarbital Cenobamate Cannabidiol Stiripentol	↓↓ ↓↓ ↓↓ ↓↓ ↓↑ ↓↑	-	
	Pharmacokinetic studies confirm that mirtazapine metabolism can be induced by carbamazepine and phenytoin			
**Bupropion**	**Information from Drug Compendia**			
	CYP2B6 induction or CYP2B6 induction/inhibition		No predicted effects	
	Carbamazepine Cenobamate Stiripentol Cannabidiol	↓↓ ↓↓ ↓↓↑↑ ↓↓↑↑	-	
	Induction of bupropion by carbamazepine and lack of effect of valproate have been confirmed in a formal pharmacokinetic study. Lack of pharmacokinetic interactions with lamotrigine has been demonstrated in an open-label study			
**Venlafaxine**	**Information from Drug Compendia**			
	CYP3A4 induction/inhibition		No predicted effects	
	Stiripentol	↓↓↑↑	-	
	Data from therapeutic drug monitoring databases have shown that levels of venlafaxine active metabolite desmethylvenlafaxine are increased by valproic acid while valproic acid levels were not affected			
**Milnacipran**	**Information from Drug Compendia**			
	No DDIs with ASMs have been reported		No predicted effects	
	In a formal pharmacokinetic study, milnacipran metabolism has been induced by carbamazepine			
**Reboxetine**	**Information from Drug Compendia**			
	CYP3A4 induction		CYP3A4 inhibition	
	Carbamazepine Phenytoin Phenobarbital	↓↓ ↓↓ ↓↓	Carbamazepine	↑↑
	Case reports have confirmed that reboxetine metabolism is induced by phenobarbital and carbamazepine			
**Duloxetine**	**Information from Drug Compendia**			
	CYP1A2 induction		No predicted effects	
	Carbamazepine Phenobarbital Stiripentol Cannabidiol	↓↓ ↓↓ ↓↑ ↓↑	-	
	No DDIs studies with ASMs in humans or case reports have been described			
Agomelatine*	CYP1A2 and, to a lesser extent, CYP2C9 and CYP2C19 induction (from SmPC)		No predicted effects	
	No DDIs studies with ASMs in humans or case reports have been described			
**Vilazodone**	**Information from Drug Compendia**			
	CYP3A4 induction or CYP3A4 induction/inhibition		No predicted effects	
	Carbamazepine Phenytoin Phenobarbital Oxcarbazepine Eslicarbazepine acetate Stiripentol	↓↓ ↓↓ ↓↓ ↓↓ ↓↓ ↓↓↑↑	-	
	Findings from a formal pharmacokinetic study have confirmed that carbamazepine induces vilazodone metabolism			
**Vortioxetine**	**Information from Drug Compendia**			
	CYP3A4 induction or CYP3A4 induction/inhibition		No predicted effects	
	Carbamazepine Phenytoin Phenobarbital Oxcarbazepine Eslicarbazepine acetate Stiripentol	↓↓ ↓↓ ↓↓ ↓ ↓ ↓↑	-	
	No DDIs studies with ASMs in humans or case reports have been described			

**Note**: All searches were performed through consultation of Medscape Interaction checker [9] and RxList [10] and in the SmPC or FDA PI of each drug. A more detailed description of clinical studies and relative references is reported in the text. There are DDIs not predicted by compendia that are clinically documented and also predicted DDIs that are not confirmed by clinical studies. Induction/inhibition means that a drug may have opposite effects on the metabolism of the victim drug.

* Drug not present in drug compendia

(↓): mild, (↓↓): moderate; (↓↓↓): severe decrease of plasma concentrations. (↑): mild, (↑↑): moderate, (↑↑↑): severe increase of plasma concentration; (↓↑): opposite effects on drug concentrations may be expected.

**Table 6:** Potential DDIs between antiseizure medications and antipsychotics and synthesis of results of clinical studies and case reports.

**Antipsychotic Drug**	**Effects of ASMs on Antipsychotic Drugs (Affected Drug)**		**Effects of Antipsychotic Drugs (Perpetrator) ** **on ASMs**	
**Typical Antipsychotics**				
**Chlorpromazine**	**Information from Drug Compendia**			
	Unspecified mechanism		No predicted effects	
	Carbamazepine Phenobarbital	↓↓ ↓↓	-	
	Findings from a pharmacokinetic study have confirmed that phenobarbital induces chlorpromazine metabolism. It has also been found that chlorpromazine increases valproic acid levels (not reported in drug compendia)			
**Trifluoperazine**	**Information from Drug Compendia**			
	CYP1A2 inhibition/induction		No predicted effects	
	Stiripentol Cannabidiol	↓↓↑↑ ↓↓↑↑	-	
	No DDIs studies with ASMs in humans or case reports have been described			
**Haloperidol**	**Information from Drug Compendia**			
	CYP3A4 induction		CYP3A4 inhibition	
	Carbamazepine Cenobamate Phenobarbital Phenytoin	↓↓↓ ↓↓↓ ↓↓↓ ↓↓↓	Carbamazepine	↑↑
	Clinical studies and case reports have demonstrated that carbamazepine reduces haloperidol levels while valproate does not have significant effects and topiramate slightly increases haloperidol levels (not reported in drug compendia)			
**Atypical Antipsychotics**				
**Ziprasidone**	**Information from Drug Compendia**			
	CYP3A4 induction		No predicted effects	
	Carbamazepine Phenytoin Phenobarbital Eslicarbazepine acetate Oxcarbazepine Rufinamide Topiramate	↓↓ ↓↓ ↓↓ ↓ ↓ ↓ ↓	-	
	Findings from a pharmacokinetic study have confirmed that carbamazepine slightly decreases ziprasidone levels			
**Lurasidone**	**Information from Drug Compendia**			
	CYP3A4 induction		No predicted effects	
	Carbamazepine Phenytoin Phenobarbital Oxcarbazepine Stiripentol	↓↓↓ ↓↓↓ ↓↓↓ ↓↓↓ ↓↓↑↑	-	
	No DDIs studies with ASMs in humans or case reports have been described			
**Pimozide**	**Information from Drug Compendia**			
	CYP3A4 and CYP1A2 induction or inhibition		No predicted effects	
	Carbamazepine Phenobarbital Phenytoin Oxcarbazepine Rufinamide Topiramate Stiripentol Cannabidiol	↓↓ ↓↓ ↓↓ ↓ ↓ ↓ ↓↓↑↑ ↓↓↑↑	-	
	No DDIs studies with ASMs in humans or case reports have been described			
**Clozapine**	**Information from Drug Compendia**			
	CYP1A2 and CYP3A4 induction		No predicted effects	
	Carbamazepine Phenytoin Phenobarbital Oxcarbazepine Eslicarbazepine acetate Topiramate Stiripentol	↓↓ ↓↓ ↓↓ ↓ ↓ ↓ ↓↓↑↑	-	
	Several clinical studies have documented that clozapine metabolism is induced by carbamazepine and phenytoin. Several clinical studies have attested an inducing effect of valproic acid on clozapine (not reported in drug compendia)			
**Olanzapine**	**Information from Drug Compendia**			
	CYP1A2 induction or CYP1A2 induction or inhibition		No predicted effects	
	Carbamazepine Phenobarbital Stiripentol Cannabidiol	↓↓ ↓↓ ↑↑ ↑↑	-	
	Formal pharmacokinetic studies have confirmed that carbamazepine induces olanzapine metabolism. Valproic acid has inducing effects, and lamotrigine has slight inhibiting effects (not reported in drug compendia)			
Quetiapine*				
	Several studies have demonstrated that carbamazepine has strong inducing effects on quetiapine metabolism. Studies assessing a DDI with valproic acid give contrasting results. Some studies have observed an inhibiting effect of valproic acid on quetiapine metabolism, while others have failed to show any effect. A slight reduction of quetiapine levels has been reported when this drug was combined with lamotrigine			
**Asenapine**	**Information from Drug Compendia**			
	CYP1A2 induction		No predicted effects	
	Carbamazepine Phenytoin Phenobarbital	↓↓ ↓↓ ↓↓	-	
	A pharmacokinetic study has shown that valproic acid affects asenapine metabolism (reduced levels of inactive metabolites) without significant changes in asenapine levels			
**Risperidone**	**Information from Drug Compendia**			
	P-gp and CYP3A4 induction or P-gp inhibition		No predicted effects	
	Carbamazepine Phenytoin Phenobarbital Stiripentol	↓↓ ↓↓ ↓↓ ↑↑	**-**	
	Several studies have demonstrated that carbamazepine induces risperidone metabolism. A pharmacokinetic study has shown that topiramate has mild inducing effects on risperidone (not reported in drug compendia). Risperidone, has been found to have mild inhibiting effects and to increase carbamazepine levels (not reported in drug compendia)			
**Aripiprazole**	**Information from Drug Compendia**			
	CYP3A4 induction or induction/inhibition		No predicted effects	
	Carbamazepine Phenytoin Phenobarbital Oxcarbazepine Rufinamide Topiramate Stirpientol	↓↓↓ ↓↓ ↓↓ ↓↓ ↓↓ ↓↓ ↓↓↑↑	-	
	Clinical studies have shown that aripiprazole metabolism is strongly influenced by carbamazepine. Clinical studies have also indicated that valproic acid has inducing effects on the metabolism of aripiprazole thus reducing its levels (not reported in drug compendia)			
**Paliperidone**	**Information from Drug Compendia**			
	CYP3A4 or P-gp induction or induction/inhibition		No predicted effects	
	Carbamazepine Phenytoin Phenobarbital Stiripentol	↓↓ ↓↓ ↓↓ ↓↑	-	
	Findings from patients have confirmed that paliperidone levels are decreased by carbamazepine while have shown that valproic acid significantly increases paliperidone levels (not reported in drug compendia)			
**Iloperidone**	**Information from Drug Compendia**			
	CYP3A4 induction		CYP3A4 inhibition	
	Carbamazepine Phenytoin Phenobarbital Oxcarbazepine Rufinamide Topiramate	↓↓ ↓↓ ↓↓ ↓↓ ↓↓ ↓↓	Carbamazepine Cannabidiol Ethosuximide Zonisamide	↑↑ ↑↑ ↑↑ ↑↑
	No DDIs studies with ASMs in humans or case reports have been described			
**Cariprazine**	**Information from Drug Compendia**			
	CYP3A4 induction or CYP3A4 induction/inhibition		No predicted effects	
	Carbamazepine Phenytoin Phenobarbital Oxcarbazepine Stiripentol	↓↓ ↓↓ ↓↓ ↓↓ ↓↓↑↑	-	
	No DDIs studies with ASMs in humans or case reports have been described			
**Brexipiprazole**	**Information from Drug Compendia**			
	CYP3A4 induction		No predicted effects	
	Carbamazepine Phenytoin Phenobarbital	↓↓ ↓↓ ↓↓	-	
	No DDIs studies with ASMs in humans or case reports have been described			

**Note**: All searches were performed through consultation of Medscape Interaction checker [9] and RxList [10] and in the SmPC or FDA PI of each drug. A more detailed description of clinical studies and relative references is reported in the text. There are DDIs not predicted by compendia that are clinically documented and also predicted DDIs that are not confirmed by clinical studies. Induction/inhibition means that a drug may have opposite effects on the metabolism of the victim drug.

*Drug not present in drug compendia.

(↓): mild, (↓↓): moderate; (↓↓↓): severe decrease of plasma concentrations. (↑): mild, (↑↑): moderate, (↑↑↑): severe increase of plasma concentration; (↓↑): opposite effects on drug concentrations may be expected.

**Table 7:** Potential DDIs between antiseizure medications and anxiolytics and a synthesis of results of clinical studies and case reports.

**Anxiolytics**	**Effects of Antiseizure Medications on Anxiolytic Drugs (Affected Drug)**	
**Diazepam**	**Information from Drug Compendia**	
	CYP3A4 and/or CYP2C19 induction or inhibition	
	Carbamazepine Phenytoin Phenobarbital Oxcarbazepine Rufinamide Felbamate Cannabidiol Stiripentol Cenobamate	↓↓ ↓↓ ↓↓ ↓ ↓ ↑↑ ↑↑ ↓↓↑↑ ↓↓↑↑
	Clinical studies have confirmed that carbamazepine increases diazepam metabolism, although without significantly decreasing its effect. In an experimental study, valproic acid resulted to inhibit diazepam metabolism and to increase its free fraction. In previous studies, it has also been reported that diazepam induces phenobarbital metabolism (not reported in drug compendia)	
**Chlordiazepoxide**	**Information from Drug Compendia**	
	CYP3A4 induction or CYP3A5 induction/inhibition	
	Carbamazepine Phenytoin Phenobarbital Stiripentol	↓↓ ↓↓ ↓↓ ↓↓↑↑
	No DDIs studies on humans or case reports in the literature	
**Clorazepate**	**Information from Drug Compendia**	
	CYP3A4 induction or CYP3A4 induction/inhibition	
	Carbamazepine Phenytoin Phenobarbital Cenobamate Stiripentol	↓↓ ↓↓ ↓↓ ↓↓ ↓↓↑↑
	No DDIs studies in humans or case reports have been described	
**Lorazepam**	**Information from Drug Compendia**	
	UGT2B7 inhibition	
	Cannabidiol	↑↑
	No DDIs studies in humans or case reports have been described	
**Alprazolam**	**Information from Drug Compendia**	
	CYP3A4 induction or CYP3A4 induction/inhibition	
	Carbamazepine Cenobamate Eslicarbazepine acetate Oxcarbazepine Phenytoin Phenobarbital Rufinamide Topiramate Stiripentol	↓↓ ↓↓ ↓↓ ↓↓ ↓↓ ↓↓ ↓↓ ↓↓ ↓↓↑↑
	In a pharmacokinetic study, carbamazepine has significantly increased alprazolam clearance	
**Buspirone**	**Information from Drug Compendia**	
	CYP3A4 induction or CYP3A4 induction/inhibition	
	Carbamazepine Cenobamate Phenytoin Phenobarbital Topiramate Oxcarbazepine Rufinamide Stiripentol	↓↓ ↓↓ ↓↓ ↓↓ ↓↓ ↓↓ ↓↓ ↓↓↑↑
	No DDIs studies in humans or case reports have been described	

**Note**: All searches were performed through consultation of Medscape Interaction checker [9] and RxList [10] and in the SmPC or FDA PI of each drug. A more detailed description of clinical studies and relative references is reported in the text.

(↓): mild, (↓↓): moderate; (↓↓↓): severe decrease of plasma concentrations. (↑): mild, (↑↑): moderate, (↑↑↑): severe increase of plasma concentration; (↓↑): opposite effects on drug concentrations may be expected.

**Table 8:** Selection of possible clinical consequences (loss of efficacy or adverse effects) caused by DDIs between antiseizure and psychiatric medications.

**Drug Interaction**	**Clinical Consequence**	**Mechanism of Interaction**	**Refs.**
Nortriptyline-carbamazepine	Inefficacy	Carbamazepine induces nortriptyline metabolism with a consequent decrease by more than 50% of its concentrations and loss of efficacy	[35]
Clomipramine-valproic acid	Status epilepticus	Inhibition of clomipramine metabolism and increased clomipramine free fraction by valproic acid coadministration may lead to toxic clomipramine levels with consequent proconvulsant effects	[29]
Clozapine-valproic acid	Myocarditis	This idiosyncratic adverse drug reaction has been associated with rapid clozapine titration. Valproic acid inhibits clozapine metabolism and increases the frequency of this reaction	[102, 103]
Quetiapine-carbamazepine	Ataxia	Quetiapine may inhibit epoxide hydrolase and/or glucuronidation of carbamazepine epoxide leading to toxic levels of the main active carbamazepine metabolite	[124]
Alprazolam-carbamazepine	Loss of anxiolytic effect	Carbamazepine induces alprazolam metabolism leading to more than 50% reduction of alprazolam concentrations and loss of efficacy	[146]

## LIST OF ABBREVIATIONS

ADs: Antidepressants
APs: Antipsychotics
ASMs: Antiseizure Medications
ATC: Anatomical Therapeutic Chemical
BRV: Brivaracetam
CBD: Cannabidiol
CBZ: Carbamazepine
CLB: Clobazam
CNB: Cenobamate
CNP: Clonazepam
DDIs: Drug-drug Interactions
ESL: Eslicarbazepine Acetate
ETS: Ethosuximide
FBM: Felbamate
GBP: Gabapentin
GVG: Vigabatrin
LCM: Lacosamide
LEV: Levetiracetam
LTG: Lamotrigine
OXC: Oxcarbazepine
PB: Phenobarbital
PER: Perampanel
PGB: Pregabalin
PHT: Phenytoin
RFN: Rufinamide
STP: Stiripentol
TPM: Topiramate
UGT: Uridine Diphosphate-glucuronosyltransferase
VPA: Valproic Acid
ZNS: Zonisamide
